# Application of nanotags and nanobodies for live cell single-molecule imaging of the Z-ring in *Escherichia coli*

**DOI:** 10.1007/s00294-023-01266-2

**Published:** 2023-04-06

**Authors:** Emma Westlund, Axel Bergenstråle, Alaska Pokhrel, Helena Chan, Ulf Skoglund, Daniel O. Daley, Bill Söderström

**Affiliations:** 1 Australian Institute for Microbiology and Infection, University of Technology Sydney, Ultimo, NSW 2007 Australia; 2 Department of Biochemistry and Biophysics, Stockholm University, 106 91 Stockholm, Sweden; 3 Structural Cellular Biology Unit, Okinawa Institute of Science and Technology, Okinawa, 904-0495 Japan

**Keywords:** ALFA-tag, Nanotags, *E. coli*, FtsZ, STED, PALM

## Abstract

Understanding where proteins are localized in a bacterial cell is essential for understanding their function and regulation. This is particularly important for proteins that are involved in cell division, which localize at the division septum and assemble into highly regulated complexes. Current knowledge of these complexes has been greatly facilitated by super-resolution imaging using fluorescent protein fusions. Herein, we demonstrate with FtsZ that single-molecule PALM images can be obtained *in-vivo* using a genetically fused nanotag (ALFA), and a corresponding nanobody fused to mEos3.2. The methodology presented is applicable to other bacterial proteins.

**Supplementary Information:**

The online version contains supplementary material available at 10.1007/s00294-023-01266-2.

## Introduction

In recent years, super-resolution fluorescence microscopy methods have taken centre stage in microbial cell biology as they can resolve cellular structures well below the diffraction limit (Xiao and Dufrene 2016). One cellular system that has benefitted greatly from these methods is the bacterial cell division machinery or the ‘divisome’. The ‘divisome’ is a complex and transient macromolecular protein assembly containing > 35 different proteins that span all compartments of the cell (Attaibi and den Blaauwen 2022; Haeusser and Margolin 2016). First to arrive at the division site is FtsZ (McQuillen and Xiao 2020), a protein that functions as a molecular foreman that guides the assembly and dynamics of other divisome proteins (den Blaauwen, et al. 2017; Erickson et al. 2010). Due to its broad conservation over many bacterial species, FtsZ could also be a potent target for new antibiotics (Kusuma et al. 2019). As such, an interest in understanding the function of FtsZ has driven the cell division field for the last 25 years (den Blaauwen et al. 2017).

One technical aspect that has hampered studies of FtsZ and other cell division proteins is that reliable labelling is an ongoing challenge for fluorescence microscopy imaging, especially in super-resolution imaging. Considerable progress has been made using N- and C-terminal fusions to fluorescent proteins (FPs), which provide a multi-wavelength tool-box for all modes of fluorescence imaging techniques (Holden 2018; Xiao and Dufrene 2016). And since FPs can be imaged in live cells, temporal changes in localization can be tracked (Holden 2018; Rowlett and Margolin 2014; Strauss et al. 2012). Major drawbacks of FP fusions are that they are large (~ 25 kDa) and that they can perturb the function of the protein they are fused to (as judged by the fact that they cannot always fully complement the phenotype of a deletion strain). As such, they often need to be expressed from plasmids and studied in the context of the native (chromosomally encoded) division protein. An unwanted side-effect of doing this is that the amount of protein in the cell is higher than normal, which can perturb the timing of division and result in an elongated cell phenotype. Eriksson and colleagues elegantly worked around this problem by exploring internal fusion points for FPs in FtsZ (Moore et al. 2017). They noted that all N- and C- terminal fusions are not functional, but that position G55:Q56 could functionally tolerate some but not all FPs. Recently similar approaches have been applied to other division proteins, e.g. FtsN (Lyu et al. 2022) and FtsA (Cameron and Margolin 2023). It should be noted that this approach is extremely time-consuming and not broadly applicable.

An alternative approach is to use fluorescently labelled antibodies to label the native protein. Here, a primary antibody binds to the protein of interest, and a fluorescent dye labelled secondary antibody is used to label the primary antibody. This approach, immunofluorescence microscopy or IFM, has been widely used but is limited by the availability and quality of antisera. Another major drawback here is the large linkage error, defined as the distance from target protein to fluorescent label (Lelek et al. 2021). The linkage error can be in the order of 30–50 nm as antibodies are relatively large and thus of comparable size to the resolving power of various super-resolution microscopy methodologies. This problem is compounded by the fact that most antibodies are polyclonal and bind to multiple epitopes on a protein of interest. These drawbacks create uncertainty about the localization of the protein being studied, which is counter-productive as super-resolution imaging system can have practical resolving powers down to a few tens of nanometres (Carrington et al. 2019). One way of circumventing this issue is to use alternative protein tags, e.g., the HaloTag (Los et al. 2008). These tags are fused to the target protein and a fluorescently labelled substrate (typically an organic dye) is added to bind to the tag. The HaloTag has been successfully used in bacterial cell division studies previously and is especially well suited for single-molecule tracking type experiments (McCausland et al. 2021). One big drawback, however, is that the Halo-tag itself is relatively large (comparable to the size of a FP) and can disrupt the function of the protein of interest.

Herein, we have instead explored the use of nanotags (NTs) and fluorescently labelled nanobodies (NBs) for labelling division proteins in *E. coli*. Specifically, we have used the ALFA-tag (Gotzke et al. 2019), a small and highly versatile protein tag that can be used for a wide range of biological applications (Akhuli et al. 2022). In bacteriology, the ALFA-tag has thus far been used in super-resolution microscopy of DNA damage repair, as well as in structural biology of cell wall synthesis (Shlosman et al. 2022; Wiktor et al. 2021), but to our knowledge never before in a dynamic live cell setting. We demonstrate that the ALFA-tag can be integrated into the genome of *E. coli* without affecting the function of the essential cell division protein FtsZ. We further demonstrate that ALFA-labelled FtsZ can be imaged by both STimulated Emission Depletion (STED) microscopy using fluorescently labelled NBs in an immunofluorescence approach, and importantly by expressing fluorescently labelled NBs from plasmids using live cell photoactivated localization microscopy (PALM). The *in-vivo* approach has a number of advantages over regular FP fusions and immunofluorescence microscopy, which are discussed.

## Methods

### Strain engineering

The FtsZ-ALFA strain was made using the CRMAGE method (Ronda et al. 2016). In short, the MG1655 strain was transformed with the pMA7CR.2.0 (amp^R^) and pZS4int-tetR (spec^R^) plasmids, then grown overnight at 37 °C (with shaking) in LB Lennox media containing 100 μg ml^−1^ ampicillin and 50 μg ml^−1^ spectinomycin. The culture was back-diluted 1:100 and grown to an OD_600_ of 0.4–0.5. Production of the λ RED β-protein was induced with 0.2% (w/v) L-arabinose for 15 min. Cells were washed three times in ice-cold water and resuspended in 400 μl of ice-cold water. A 50 μl aliquot of the cells was mixed with 1 μl of 5 μM CRMAGE oligo (Integrated DNA Technologies, USA) and 250 ng of the pMAZ-SK (kana^R^) plasmid (containing the sgRNA for the native *ftsZ* locus) and transferred to an electroporation cuvette with a 1 mm gap. Cells were electroporated at 1.8 kV and then mixed with 950 μl of LB media containing 100 μg/mL ampicillin and 50 μg ml^−1^ spectinomycin. After a 1-h recovery at 37 °C with shaking, 50 μg ml^−1^ of kanamycin was added, and cells were incubated for 2 h at 37 °C. The Cas9 protein was induced by the addition of 200 ng ml^−1^ anhydrotetracycline, and the culture was further incubated at 37 °C (with shaking) overnight. A dilution series was plated on LB agar containing 100 μg ml^−1^ ampicillin, 50 μg ml^−1^ spectinomycin and 50 μg ml^−1^ of kanamycin. Colonies were screened by diagnostic PCR to identify recombinant strains. The sequence of the CRMAGE oligo is listed in Supplementary Table S1. The pMAZ-SK plasmid containing the sgRNA was constructed using the in vivo cloning approach (Bubeck, et al. 1993; Garcia-Nafria et al. 2016; Jones and Howard 1991). In short, the plasmid backbone was amplified by PCR using primers P9 and P10 and the Q5 DNA polymerase (New England Biolabs, USA, NEB #M0491). These primers were obtained from Eurofins Genomics (Germany) and are listed in Supplementary Table S1. The plasmid was sequenced verified by Eurofins Genomics (Germany).

The engineered FtsZ-ALFA strain was subsequently cured from the pMAZ-SK plasmid as previously described with minor modifications (Ronda et al. 2016). In short, cells were grown overnight then washed twice with fresh LB and back diluted in fresh LB with anhydrotetracycline 200 ng ml^−1^ and 0.2% (w/v) of L-Rhamnose and grown for 24 h. Cultures were plated on Amp/Spect (100 μg ml^−1^ ampicillin, 50 μg ml^−1^ spectinomycin) and Kan plates (50 μg ml^−1^ Kanamycin). The final cured strain MG1655 (FtsZ-ALFA(G55:Q56)) was denoted AB003.

### Plasmid construction

The expression plasmid pBAD/HisB-mEos3.2-α-ALFA (kana^R^) was constructed using the pBAD/HisB(TIR^EVOL^)-mEos3.2 (amp^R^) backbone (Addgene plasmid #189,720; (Shilling et al. 2022)). Initially, pBAD/HisB(TIR^EVOL^)-mEos3.2 (amp^R^) was linearised by PCR using the primers P1 and P2. The coding sequence for the α-ALFA NB was amplified by PCR from the mEGFP-NbALFA plasmid (Addgene plasmid #159,986; (Jin et al. 2021)) using the primers P3 and P4. This PCR product contained 5′ and 3′ extensions (19 and 18 bps, respectively) that overlapped with the desired integration site in the pBAD/HisB(TIR^EVOL^)-mEos3.2 (amp^R^) backbone so that they could be ligated using the in vivo cloning approach (Bubeck et al. 1993; Garcia-Nafria et al. 2016; Jones and Howard 1991). In short, this required that the PCR products were treated with *Dpn*I enzyme for 60 min at 37 °C, then transformed into MC1061 strain using a standard heat shock protocol. The final clone was called pBAD/HisB-mEos3.2-α-ALFA (amp^R^). In a second step, the Tn3.17 fragment containing the coding sequence for ß-lactamase (and conferring resistance to ß-lactam antibiotics; amp^R^) was replaced with the Tn903.1 fragment containing the aminoglycoside 3′ phosphatase (and conferring resistance to aminoglycoside antibiotics; kana^R^). pBAD/HisB-mEos3.2-α-ALFA (amp^R^) was linearised by PCR using the primers P5 and P6. The coding sequence for the Tn903.1 was amplified by PCR from the pET28 (T7p^CONS^/TIR2)-sfGFP plasmid (Addgene plasmid #154,464;(Shilling et al. 2020)) using the primers P7 and P8. This PCR product contained 5′ and 3′ extensions (15 and 18 bps, respectively) that overlapped with the integration site in the pBAD/HisB-mEos3.2- α -ALFA backbone so that they could be ligated using the in vivo cloning approach (Bubeck et al. 1993; Garcia-Nafria et al. 2016; Jones and Howard 1991). The final clone was called pBAD/HisB-mEos3.2- α -ALFA (kana^R^). PCR was carried out with the Q5 DNA polymerase (New England Biolabs, USA, NEB #M0491).

Finally, to construct pBAD/HisB-sfGFP-α-ALFA (kana^R^), we exchanged the coding sequence of mEos3.2 for that of sfGFP in the pBAD/HisB-mEos3.2-α -ALFA (kana^R^) plasmid using primers P11–P14. The sfGFP coding sequence was PCR amplified from pGI5 plasmid (Iosifidis and Duggin 2020), using primers P13 and P14. The pBAD-mEos3.2-αALFA (kana^R^) plasmid backbone was PCR amplified using primers P11 and P12, before combining the PCR products using Gibson assembly using NEBuilder® HiFi DNA Assembly Master Mix (New England Biolabs, USA, NEB #E2621L). The assembled product was then transformed into *E. coli* DH5α chemical competent cells before selecting the positive colonies and isolating the plasmid. The isolated pBAD/HisB-sfGFP- α -ALFA (kana^R^) plasmid was sequenced to confirm the correct integration of sfGFP coding sequence and transformed into *E. coli* MG1655 FtsZ-ALFA strain for further analysis.

Primers were obtained from either Eurofins Genomics (Germany) or Integrated DNA Technologies, Inc. and are listed in Supplementary Table S1. Plasmids were sequenced verified by sanger sequencing from Eurofins Genomics (Germany) or Australian Genome Research Facility (AGRF), Sydney.

### Bacterial growth and plasmid expression

Pre-cultures of AB003 with or without pBAD/HisB-mEos3.2-α-ALFA were grown overnight in 20 ml of LB with appropriate antibiotics (Ampicillin 100 μg ml^−1^, Kanamycin 50 μg ml^−1^) at 30 °C. The following morning the cultures were back-diluted 1:50 in LB and incubated at 30 °C to OD_600_ 0.3 (~ 2 h). AB003 cells without the plasmid were processed for immunofluorescence and STED (see ‘Cell fixation and nanobody labelling for STED’). Cultures of AB003 transformed with pBAD/HisB-mEos3.2-α-ALFA were pelleted and resuspended in fresh M9 minimal media supplemented with 0.2% casamino acids, 1 mM MgSO_4_, and 0.00005% arabinose (to initiate production mEos3.2-α-ALFA production) and grown at 30 °C for an additional two hours.

Four μl of cell culture were added to an agarose pad and taken for live cell imaging.

### Cell fixation and nanobody labelling for STED

Cells were fixed and immunodecorated as described previously (Söderström et al. 2018b). Briefly, 900 μl of ice-cold methanol were added to 100 μl of cell culture (OD_600_ ~ 0.2–0.5) and incubated for 5 min on ice. Cells were harvested by centrifugation and resuspended in 100 μl of 90% (*v/v*) ice-cold methanol. For nanobody labelling of cells, roughly 20 μl of cell suspension applied on a cover glass coated with Poly-L-Lysine or on agarose micron-hole beds and left for ~ 2 min before excess culture was removed. After drying, the cells were treated with lysozyme solution (0.2 mg/mL lysozyme, 25 mM Tris–HCl pH 8.0, 50 mM glucose, 10 mM EDTA) for 7–8 min and then rinsed with PBSTS [140 mM NaCl, 2 mM KCl, 8 mM Na_2_HPO_4_, 0.05% (v/v) Tween 20, 20% (w/v) sucrose], before they were treated with methanol for 1 min and acetone for 1 min and dried. Fixed cells were incubated with 3% (w/v) bovine serum albumin in PBSTS for at least 15 min. Thereafter the cells were washed with PBSTS and incubated with fluorescently labelled nanobodies (*i.e.* α-ALFA-ATTO488; cat. no. N1502-At488-L, NanoTag Biotechnologies, gift from Dr. Hansjörg Götzke) for 2–3 h at RT. Cells on cover glasses were placed on glass slides with a drop of ProLong Gold antifade as mounting medium and left overnight to harden before imaging, while cells in agarose micron-hole beds were covered with a pre-cleaned cover glass.

### Imaging

STED images were acquired on a Leica TCS SP8 STED 3X system, using a HC PL Apo 100 × oil immersion objective with NA 1.40. ATTO488 was excited using a white excitation laser operated at 488 nm, and STED was achieved using a 592 nm laser line. The total intensity of the depletion laser was in the order of 20–500 MW/cm^2^ for all STED imaging. To achieve time gated STED was a detection time-delay of 0.9–2.5 ns applied. The final pixel size was 13 nm and scanning speed 600 Hz. The pinhole size was varied between 0.4 and 0.9 AU; a smaller pin-hole when imaging cells standing trapped in micron holes and larger when imaging cells lying flat on agarose pads.

Live cell PALM imaging was performed on a Nikon (Ti2-E) N-STORM v5 with NIS v.5.30 using a 100 × 1.49 NA oil objective, operated in quasi-TIRF mode to increase the signal to noise ratio. A stage-top environmental chamber (Oko lab) was used for temperature control, imaging was performed at 37 °C for live cell and time-lapse imaging. Cover glass slides were washed with 80% EtOH, air dried, cleaned for at least 3 min with a plasma cleaner (Harrick plasma, PDC-23G) and used within 15 min of cleaning. Prior to single-molecule acquisition, the green state of mEos3.2 was excited by a 488 nm laser to acquire an epifluorescence image using a FITC emission filter cube. For single-molecule acquisition, mEos3.2 was photoconverted to its red state continuously by a 405 nm laser with increasing working power ranging between 0.1 and 5 W cm^−2^. As readout, mEos3.2 was excited by a 561 nm laser line operating at an average power between 1 and 2 kW/cm^2^. The emission was collected by a quad band (Quad405/488/561/647 filter dual cSTORM). The exposure time was 20 ms and ~ 3000–4000 images were typically acquired for each set of images. Drift correction during image acquisition was minimised using the integrated PFS4 (Perfect Focus System). The relatively short PALM image acquisition time (50 Hz) combined with that the overall system drift was consistently less than one half pixel over the series acquisition time, therefore no fiducial markers were needed (Supplementary Fig. 1). Individual images were captured using a sCMOS Flash 4.0 v3 (Hamamatsu) camera using a final pixel size of 32.4 nm.

### Image analysis

STED images were deconvoluted using Huygens Professional deconvolution software (SVI, the Netherlands). The resolution in our STED images was ~ 50 nm (Söderström et al. 2018b). Single-molecule raw data were analysed using the Fiji plug-in ThunderSTORM (Ovesny et al. 2014). Super resolution single-molecule images were rendered using Gaussian blur of 20 nm for visualization. Z-ring widths in cells lying down were extracted from fluorescence intensity profile line traces over midcell. A Gaussian distribution was fitted to the intensity profiles in order to extract the Full Width at Half Maximum (FWHM). Radii from cells trapped standing were estimated by manually fitting a circle over the fluorescence traces in Fiji. Statistics and graphs were generated using custom MATLAB scripts, Origin9 Pro and PlotsOfData (Postma and Goedhart 2019).

### Cell length measurements

Cells of WT, FtsZ-ALFA, FtsZ-ALFA/mEos3.2-α-ALFA and FtsZ-ALFA/sfGFP-α-ALFA were harvested from growth cultures by centrifugation. An aliquot (2 μl) was placed on an agarose (1.5% w/w) pad and directly imaged under bright-field illumination. Cell lengths of ~ 200 cells from each strain were determined using MicrobeJ (Ducret et al. 2016). Statistics and graphs were generated using custom MATLAB scripts, Origin9 Pro and PlotsOfData (Postma and Goedhart 2019).

### Western blotting

Cell extracts from a volume corresponding to 0.2 OD_600_ units were collected for different strains. The extracts were suspended in loading buffer and resolved by sodium dodecyl sulphate polyacrylamide gel electrophoresis (SDS-PAGE). Proteins were transferred to nitrocellulose membranes using a semi-dry Turbo Transfer-Blot apparatus (Bio-Rad). The membranes were blocked in 5% (w/v) milk and probed with antisera to FtsZ [1:4000] (Agrisera, Sweden).

## Results

**Fig. 1:**
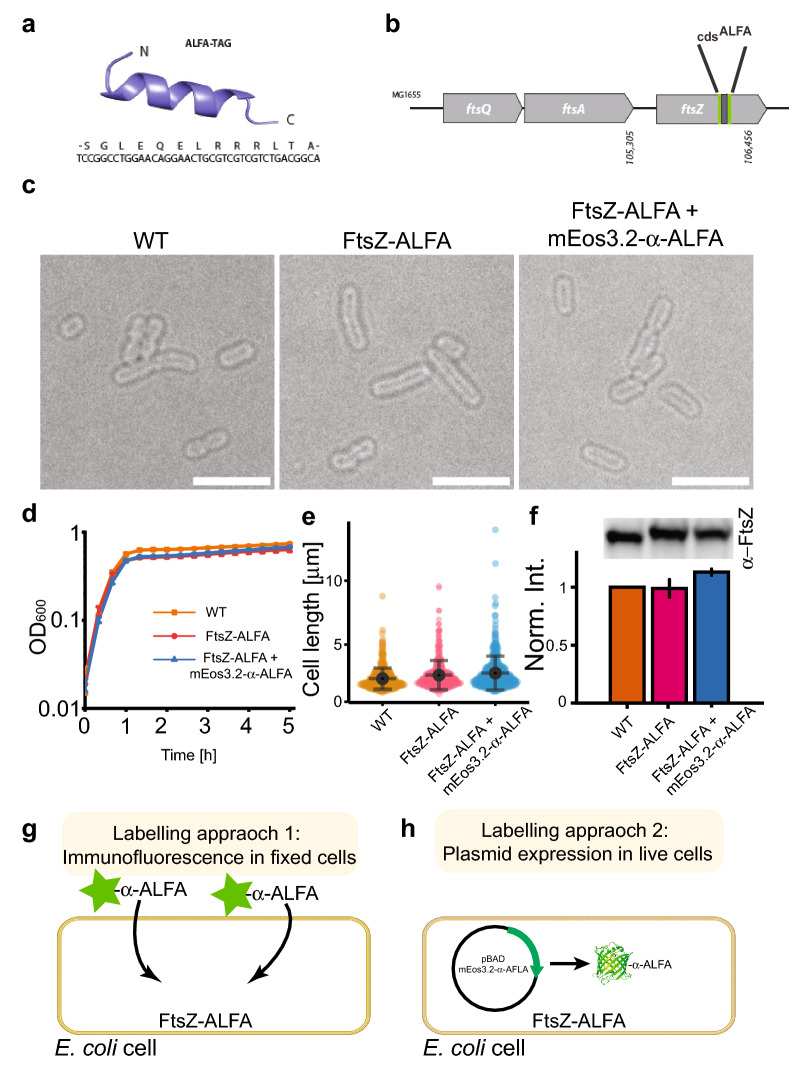
Cell viability of the engineered FtsZ-ALFA strain and labelling approaches. **a** Structure and sequence of the ALFA-tag (Gotzke et al. 2019). **b** Locus on the chromosome in strain MG1655 where the ALFA-tag is incorporated. **c** Bright field images of strains used in this study. No morphological differences are noticeable. Scale bars = 5 m. **d** Growth curves of strains used (*n* = 3 for each strain). **e** Cell length measurements. WT = 2.34 ± 0.83 μm, FtsZ-ALFA = 2.61 ± 1.16 μm, FtsZ-ALFA + mEos3.2-α-ALFA = 2.78 ± 1.33 μm (*n* = 214 for each strain). Mean ± S.D. **f** Quantitative western blotting indicated that neither the ALFA-tag nor the additional expression of mEos3.2-α-ALFA altered the overall expression of FtsZ to any larger degree. WT = 1,FtsZ-ALFA = 0.99 ± 0.08,FtsZ-ALFA + mEos3.2-α-ALFA = 1.13 ± 0.03. Mean ± S.D. *n* = 3. Full length WB shown in Supplementary Fig. 3. **g** Approach 1: immunofluorescence labelling. Cells were fixed, membranes permeabilized followed by labelling with ATTO488 tagged α-ALFA nanobodies recognizing the ALFA-tag. **h** Approach 2: plasmid expression in live cells. Cells were transformed with a plasmid encoding for mEos3.2-α-ALFA nanobody. In both approaches, FtsZ-ALFA is labelled with fluorescently tagged nanobodies and imaged using super-resolution approaches (Figs. 2 and 4)

Our goals in this study were (1) to determine whether an essential and normally difficult to tag *E. coli* cell division protein would remain functional when fused to a NT, and (2) if it could be imaged using super-resolution microscopy. The ALFA-tag is 39 nucleotides long and encodes a 13 amino acid peptide that forms an α-helix (Fig. 1a) (Gotzke et al. 2019). The coding sequence for ALFA was successfully inserted into the chromosomal copy of *ftsZ* in the *E. coli* strain MG1655 using CRISPR Optimized MAGE Recombineering (CRMAGE) (Ronda et al. 2016). It was possible to integrate the coding sequence for ALFA in the region of *ftsZ* corresponding to G55:Q56 by using flanking sequences (GSTLE and LEGST) (Fig. 1b). Attempts to insert the coding sequence for ALFA at the same position (G55:Q56) but without the flanking sequences, or at the C-terminus of FtsZ were not successful, suggesting that the designs were not functionally tolerated (as noted previously with FPs (Moore et al. 2017)). The strain, herein denoted FtsZ-ALFA, had no visible phenotype when visualized by light microscopy (Fig. 1c). The cell lengths were indistinguishable from the wildtype parent strain (Fig. 1e), as was the cell growth (Fig. 1d). Quantitative western blotting of cells harvested in the exponential phase indicated that levels of FtsZ in wild type and the FtsZ-ALFA strain were comparable (Fig. 1f). Taken together, these data indicate that ALFA can be functionally fused to FtsZ in a non-disruptive way. To determine if this tagging strategy is suitable for super resolution imaging, we used two orthogonal labelling approaches: immunofluorescence labelling in fixed cells (Fig. 1g), and plasmid expression of α-ALFA fused to mEos3.2 (a photoconvertible FP) in live cells (Fig. 1h). We then visualised the tagged proteins with two standard super-resolution techniques: STimulated Emission Depletion (STED) microscopy (Hein et al. 2008; Vicidomini et al. 2011) and single-molecule photoactivated localization microscopy (PALM) (Betzig et al. 2006; Greenfield et al. 2009).

### STED and immunofluorescence

**Fig. 2:**
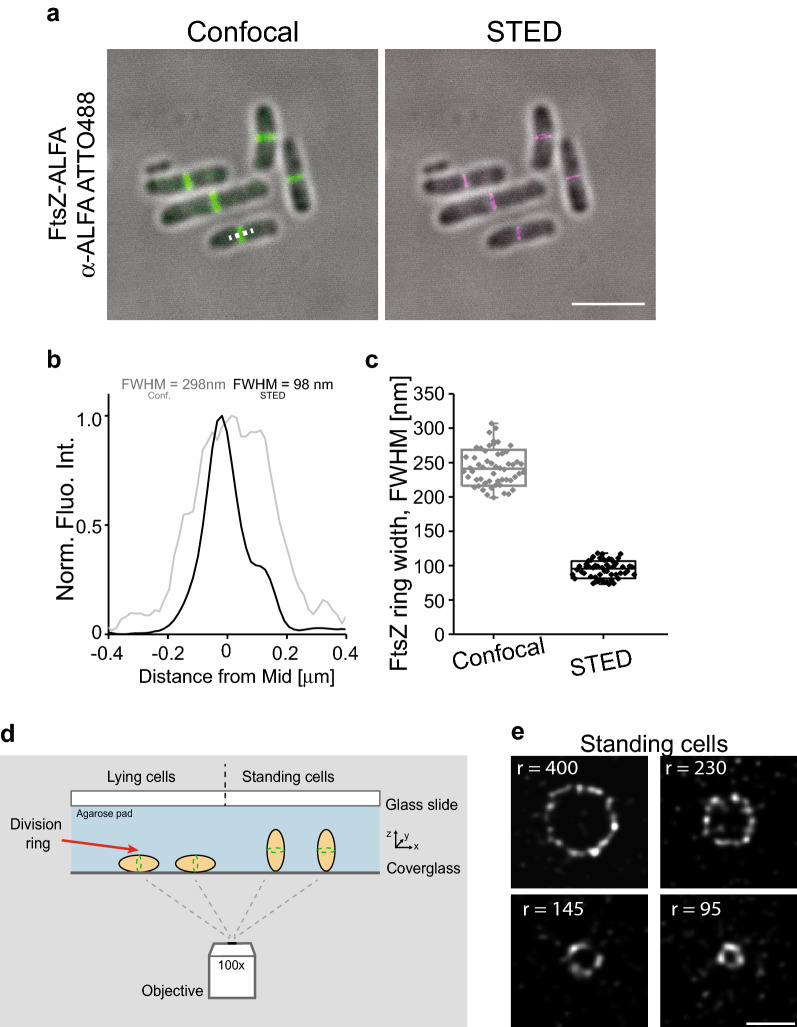
Super-resolution STED imaging of FtsZ-ALFA using an α-ALFA nanobody labelled with ATTO488. **a** Fixed cells immunolabelled and imaged using confocal and STED microscopy. Fluorescence signal overlayed with bright field images. Scale bar = 4 μm. **b** Longitudinal width (FWHM) of a representative FtsZ ring. Conf = Confocal. **c** Apparent widths (FWHM) were extracted from line scans as indicated by the white dotted line in the lower cells in the confocal image (**A**). The mean widths were 243 ± 26 nm (confocal) and 93 ± 12 nm (STED) (*n* = 54). Boxes indicate S.D., midline is the mean and whiskers incorporate 1-99% of the data range. **d** Schematic representation of cells lying on an agarose pad and trapped in a vertical position. **e** STED imaging of FtsZ-ALFA cells labelled with α-ALFA-ATTO488 trapped in a vertical position in micron holes. Representative FtsZ rings of various radii. *r* indicate radius. Scale bar = 500 nm

As a first approach, we used an established immunofluorescence methodology to label FtsZ-ALFA. Cells were fixed with formaldehyde, membranes were permeabilized using standard protocols, and FtsZ-ALFA was labelled with α-ALFA-ATTO488 nanobodies and imaged by confocal fluorescence microscopy and super-resolution STED (Vicidomini et al. 2013). As anticipated, most elongated cells had a visible FtsZ band at the future division site (Fig. 2a) (Sun and Margolin 1998). Images and quantitative measurements of the FWHM indicated that the resolution improvement by STED over conventional confocal imaging was approximately three-fold (Fig. 2b). The average width of the FtsZ-ALFA bands observed from the STED images was determined to be 94 ± 12 nm compared to 243 ± 26 nm for confocal (Fig. 2c). These STED values were slightly lower than those previously reported using STED on IFM labelled FtsZ in *E. coli* (~ 110 nm) (Söderström et al. 2018b), possibly because the linkage error was reduced using the nanotag approach. FtsZ forms filaments that treadmill around the division septum (Bisson-Filho et al. 2017; Yang et al. 2017). Under the super-resolution light microscope, these FtsZ filaments resemble elongated clusters (Fu et al. 2010; Lyu et al. 2022; Söderström et al. 2018b). Here we visualised FtsZ-ALFA rings in cells trapped in a vertical position using a micron-cage approach (Fig. 2d) (Söderström et al. 2018b). As expected, clearly resolvable FtsZ-ALFA clusters were distributed around the division septum and pseudo-time courses of fixed cells could be constructed from FtsZ-ALFA rings at various stages of constriction, with radii less than 100 nm (Fig. 2e).

### Live cell PALM using intracellular expressed fluorescent nanobodies

A limitation of the immunofluorescence approach is that cells are fixed, so it is not possible to follow real dynamics of FtsZ-ALFA over time. To circumvent this problem, we transformed the FtsZ-ALFA strain with engineered plasmids encoding for α-ALFA nanobody fused to sfGFP or the photoconvertible mEos3.2 (a FP compatible with single-molecule imaging). The main benefits of this approach are that (1) we could image and follow live cells throughout division, (2) we could control the amount of α-ALFA (so that not all FtsZ-ALFA molecules were labelled) and (3) we could induce α-ALFA when required. FtsZ-ALFA cells transformed with α-ALFA-GFP (Supplementary Fig. 2) showed localization patterns and dynamics of similar to those of FtsZ-GFP described multiple times previously (Buss et al. 2013; Ma et al. 1996; Mannik et al. 2018; Söderström et al. 2014; Sun and Margolin 1998).

**Fig. 3:**
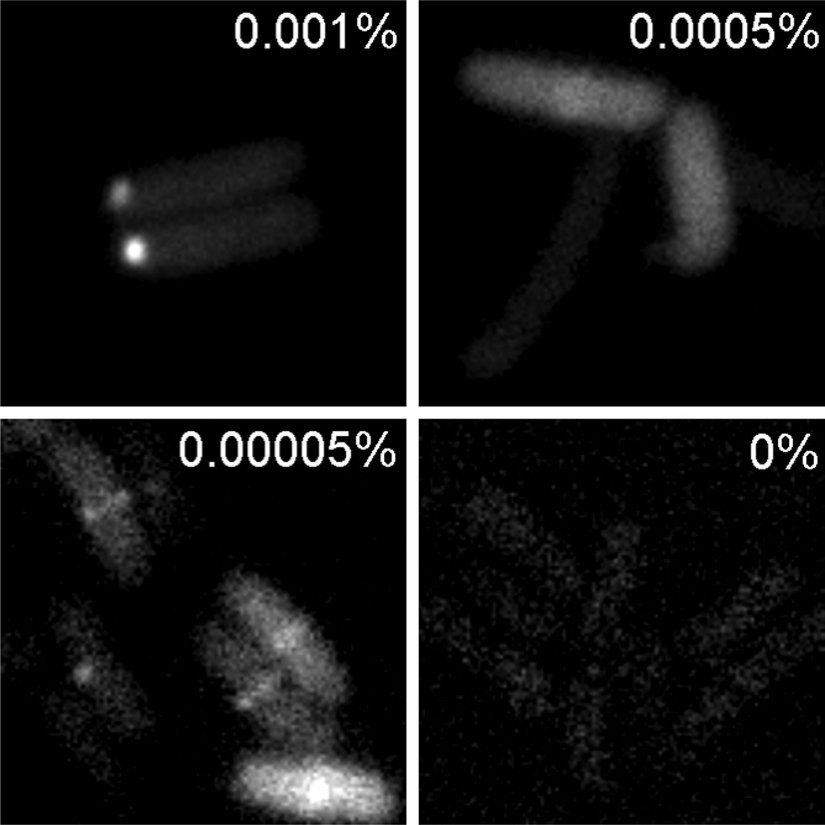
Efficient FtsZ-ALFA/mEos3.2-α-ALFA ring detection is concentration dependent. Epifluorescence imaging on FtsZ-ALFA tagged with mEos3.2-α-ALFA in live E. coli cells. mEos3.2 was excited and captured in the green state using a 488-laser line. Images of various conditions with different Arabinose concentrations, ranging from 0.001% to 0%. Too high induction (0.001% and above) produced large bright protein aggregates at the poles, 0.0005% produced too high background, while no induction resulted in essentially no fluorescence signal. As clearly can be seen, under our experimental conditions, 0.00005% Arabinose gave best results. Scale bar = 2 μm

**Fig. 4:**
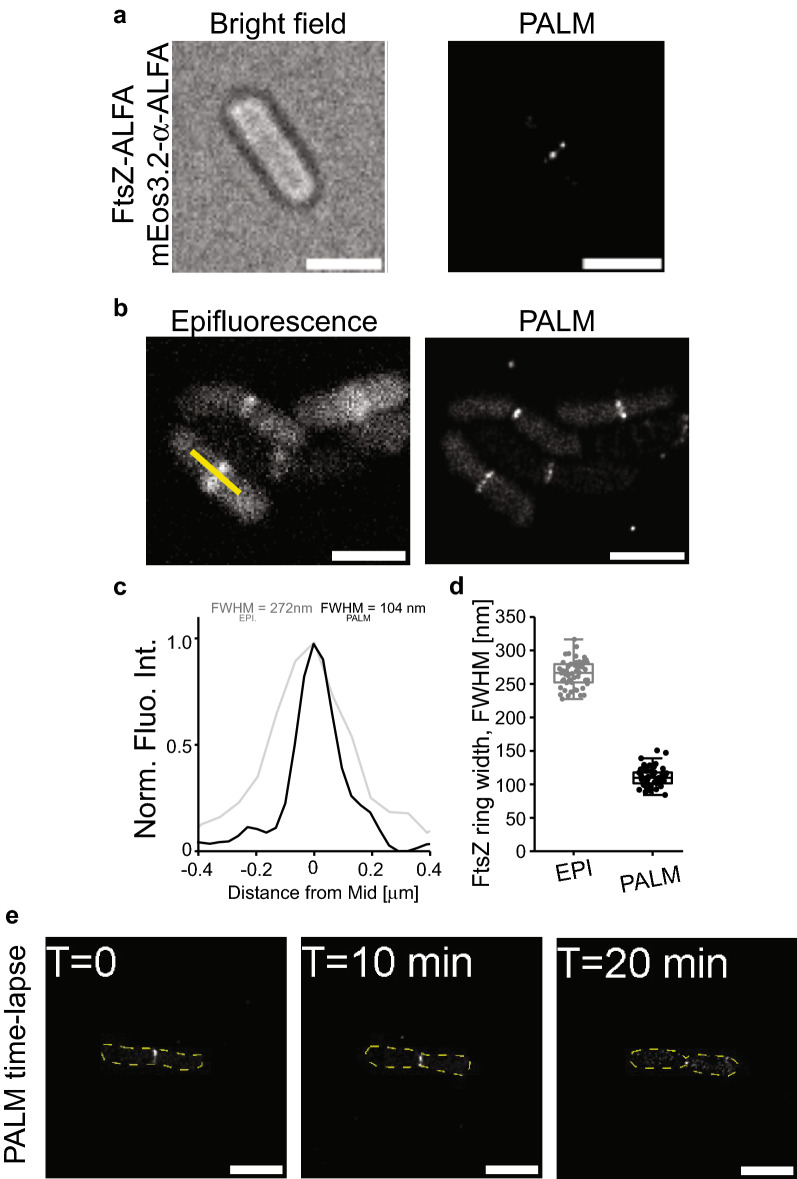
Live cell single-molecule PALM imaging of FtsZ-ALFA using a co-expressed mEos3.2-α-ALFA nanobody. **a** A typical live cell imaged by bright field optics and using PALM. **b** Side-by-side comparison of FtsZ-ALFA rings using Epifluorescence and PALM. **c** Longitudinal width (FWHM) of a representative FtsZ-ALFA ring as determined by fitting a gaussian to fluorescence traces generated drawn over the FtsZ-ALFA rings (e.g., yellow line in (B)). *EPI *Epifluorescence. **d** Apparent widths (FWHM) from line scans as indicated by the yellow line in the lower cells in the epifluorescence image (B). The mean widths were 266 ± 22 nm (epifluorescence) and 110 ± 11 nm (PALM) (*n =* 109). Boxes indicate S.D., midline is the mean and whiskers incorporate 1–95% of the data range. **e**, Images from a typical time-lapse PALM sequence. Z-ring radius is clearly decreasing over time. Scale bars = 2 μm

In PALM, the localization of single molecules from stochastically photoswitched or photoactivated fluorescent proteins are determined at sub-pixel resolution in a large series of sequential images from which a composite super-resolution image is created (Betzig, et al. 2006; Coltharp and Xiao 2012; Lelek et al. 2021). After titrating the amount of mEos3.2-α-ALFA, it was possible to identify conditions where our experimental single-molecule approach showed clear FtsZ accumulation at midcell without affecting cell morphology or growth (Figs. 3 and 4a).

Again, quantitative measurements of the FWHM indicated a substantial resolution improvement over diffraction limited imaging (Fig. 4b–c). These Z-rings had an average lateral width of 110 ± 11 nm (*n* = 109) (Fig. 4c), again similar to previously reported values using live cell PALM on FtsZ-mEos (Coltharp et al. 2016; Fu et al. 2010). One important consideration with this approach is that the expression of mEos3.2-α-ALFA needs to be optimised by titration. If expression is too low, it will not be possible to detect the ALFA nanotag, and if the expression is too high, the background fluorescence will be too high (as the mEos3.2-α-ALFA is fluorescent even when it is not bound to the ALFA nanotag) (Fig. 3) (de Beer and Giepmans 2020). This latter problem is unique to live cell imaging as it is not possible to wash away unbound mEos3.2-α-ALFA (as it is with IFM approaches).

The main advantage of this approach over IFM approaches is arguably that cells are imaged live and thus dynamic processes can be followed in super-resolution and avoid artifacts from fixing cells. To capture FtsZ dynamics at single-molecule resolution, we imaged cells on agarose pads over time. We acquired images every 10 min as it was not feasible to decrease the interval between imaging due to the limited pool of mEos3.2-α-ALFA molecules. Time-lapse imaging showed dynamic FtsZ-rings with decreasing radius over time (Fig. 4d).

## Conclusions

In summary, here we have demonstrated that nanotag/nanobody labelling approaches can be used to image bacterial cell division proteins at super resolution. Initially, FtsZ-ALFA rings were visualised in fixed cells using an immunofluorescence microscopy (IFM) protocol and super-resolution STED imaging (see also Söderström et al. 2018a, b; Strauss et al. 2012).

In a second step, we also demonstrated the feasibility of nanobody labelling in live bacteria for the first-time using plasmid encoded α-ALFA nanobodies fused to fluorescent proteins sfGFP or mEos3.2. In the latter, this allowed us to use a single-molecule PALM imaging approach. This approach allowed protein specific cell division dynamics at single-molecule resolution to be monitored over time. The advantages of our live cell nanobody approach over conventional fluorescent protein fusions are two-fold: (1) the ALFA-tags are small and are therefore less likely to interfere with normal protein function, and (2) the generation of the nanobody itself is controllable by induction meaning that its production can be restricted to biologically relevant time-points. Two limitations for this approach are also noted: (1) the expression levels of the nanobody fused to the need to be carefully optimised so that the signal to noise ratio is good, and (2) multicolour experiments are not currently feasible as only one plasmid encoded nanobody (the α-ALFA) is currently available. There are, however, several nanotags (e.g., the SPOT-tag (Virant et al. 2018), so this issue may be resolved in the future.

As a general approach, we believe that nano-tagging can easily be translated to a wide range of other events in bacteria and aid others looking to study time dependent processes at single-molecule resolution in systems otherwise challenging to label using standard approaches.

## Supplementary Information

Below is the link to the electronic supplementary material.

Supplementary file1 (DOCX 702 KB)

## Data Availability

The data supporting the findings of the study are available in this article, or from the corresponding authors upon request.
