# Supplementary material for: Application of nanotags and nanobodies for live cell single-molecule imaging of the Z-ring in Escherichia coli
Source: Curr Genet. 2023 Apr 6;69(2-3):153–63. doi: 10.1007/s00294-023-01266-2 (PMC10163087; doi:10.1007/s00294-023-01266-2)
Supplement: Supplementary file 1 — Supplementary file1 (DOCX 702 KB) [file 294_2023_1266_MOESM1_ESM.docx]

Supplementary Information for

**Application of nanotags and nanobodies for live cell single-molecule imaging**

**of the Z-ring in *Escherichia coli***

Emma Westlund^1,#^, Axel Bergenstråle ^2,#^, Alaska Pokhrel^1^,
Helena Chan^3^, Ulf Skoglund^3^, Daniel O. Daley^2,^* and Bill Söderström^1,^*

^1^ Australian Institute for Microbiology and Infection, University of Technology Sydney, Ultimo, NSW, 2007 Australia.

^2^ Department of Biochemistry and Biophysics, Stockholm University, SE-106 91 Stockholm, Sweden.

^3^ Structural Cellular Biology Unit, Okinawa Institute of Science and Technology, 904-0495 Okinawa, Japan^.^

**
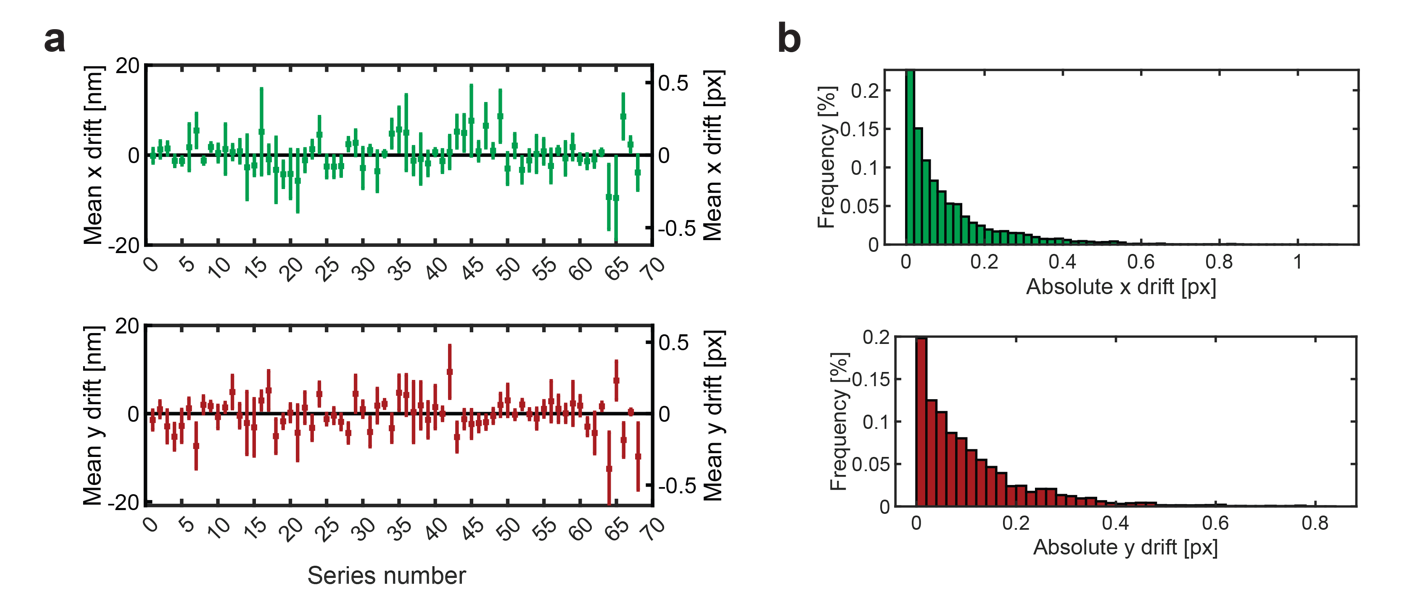
**

**Supplementary figure 1. Mean drift during single-molecule image acquisition.**

**a**, The mean drift ( ± standard deviation) over the full series in the x- and y-direction for randomly chosen image series during PALM imaging of mEos3.2. **b**, Frequency of drift between two subsequent images indicated in pixels. The drift was less than 0.56 pixels for 99% of all images in the data set (in both x and y direction). Bins are 0.02 pixels.

**
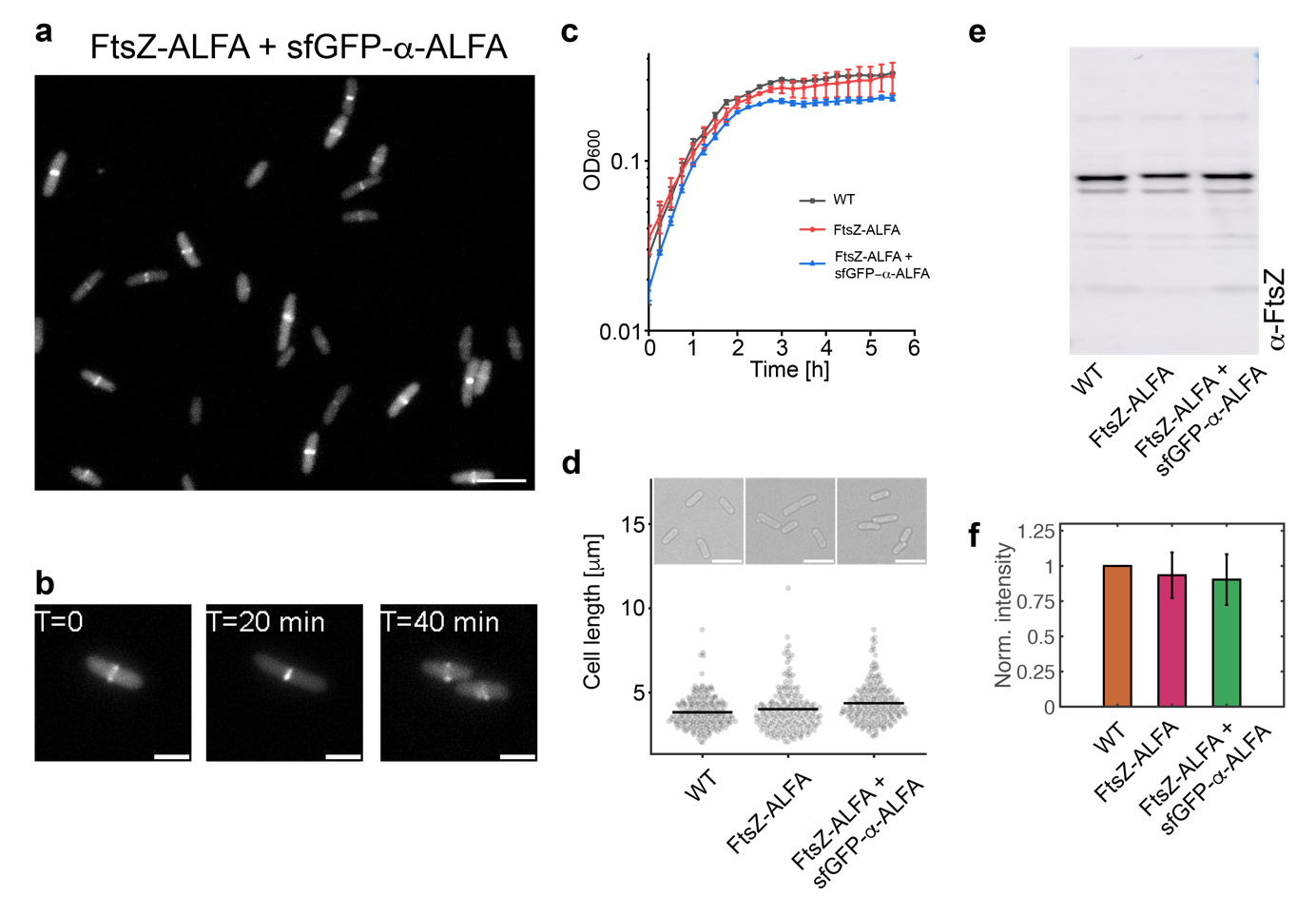
**

**Supplementary figure 2. FtsZ-ALFA labelled with sfGFP-α-ALFA in live cells.**

The FtsZ-ALFA strain (AB003) was transformed with a plasmid encoding
sfGFP-α-ALFA and grown in LB at 30 °C. Production of sfGFP-α-ALFA was initiated by adding 0.0001% (w/v) L-arabinose to the cultures. The cultures were left to grow for one hour under constant shaking at 200 r.p.m., before harvesting the cells. 4 μl of cell culture was spread on a M9 minimal media + 1.5% (w/v) agarose pad, placed in a stage-top environmental chamber operated at 37 °C mounted on a Nikon TiE2 N-STORM microscope and imaged using epi-fluorescence illumination. Growth and lengths were minimally affected by the addition and production of the sfGFP-α-ALFA plasmid. **a**, Representative image of live FtsZ-ALFA cells expressing sfGFP-α-ALFA. Scale bar = 4 μm. **b**, Still images from a time-lapse series showing FtsZ-ALFA/sfGFP-α-ALFA dynamics over time. Images were acquired every 10 minutes; total imaging time was 60 minutes. Given the photon budget available, the interval between images could be reduced to achieve better time resolution when desired. Scale bar = 2 μm. **c**, Growth curves. **d**, Cell lengths. WT = 3.84 ± 0.97 μm, FtsZ-ALFA = 4.02 ± 1.6 μm, FtsZ-ALFA/sfGFP-α-ALFA = 4.37 ± 1.17 μm (n = 198 for each strain). Inset, representative bright field images of each strain. Scale bar 4 μm. **e**, Western blot indicating FtsZ-ALFA levels were close to native FtsZ levels. **f**, Quantification of cellular FtsZ levels in different strains: WT = 100%, FtsZ-ALFA = 93 ± 16% and FtsZ-ALFA/sfGFP-α-ALFA = 90 ± 18% (n = 3).

**
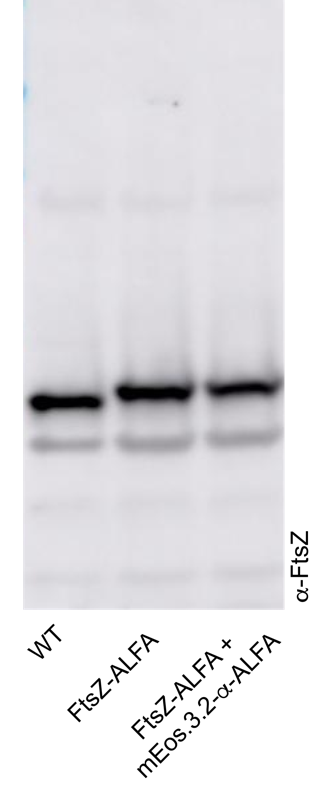
**

**Supplementary figure 3. Uncropped western blot from Figure 1.**

Figure shows the full length of the cropped western blot in Figure 1.

**Supplementary Table S1. Primers and CRMAGE oligonucleotide used.**

Primers

P1 pBAD.fwd 5’-GAATTCGAAGCTTGGCTGTTTTGGCGG-3’

P2 mEos.rev 5’-TCGTCTGGCATTGTCAGGCAATCC-3’

P3 NbALFA.fwd 5’-GCCTGACAATGCCAGACGAGGCAGTGGCAGTGGCAGTGGAG-3’

P4. NbALFA.rev 5’-CAGCCAAGCTTCGAATTCTTAGCTGCTCACAGTCACTTGGGTGCC-3’

P5 Bla loop out primer 1 5’-AGAGTTTGTAGAAACGCAAAAAG-3’

P6 Bla loop out primer 2 5’-CTGTCAGACCAAGTTTACTC-3’

P7 Aph intro primer 1 5’-GTTTCTACAAACTCTCATGAACAATAAAACTGTCTG -3

P8 Aph intro primer 2 5’-CTGACAGCTTAGAAAAACTCATCGAGCATCAAATG-3’

P9 FtsZ-crRNA-Fwd 5’- AGCGCTGCGTAAAACAGGTTTTAGAGCTAGAAATAGCAAG -3’

P10 FtsZ-crRNA-Rev 5’- TTTACGCAGCGCTTGTGTGCTCAGTATCTCTATCACTG -3’

P11 pBad-mEos3.2-αALFA Fwd 5’- GGCAGTGGCAGTGGCAGT-3’

P12 pBad-mEos3.2-αALFA Rev 5’- AGATCTCGAGCTCGGATCCTTATCGTC -3’

P13 sfGFP amplification primer 1 5’- GATGACGATAAGGATCCGAGCTCGAGATCTGGTAAA
 GGTGAAGAACTGTTCACC -3’

P14 sfGFP amplification primer 2 5’- CTGAACTTCTCCACTGCCACTGCCACT
 GCCTTTGTAGAGTTCATCCATGCCGTG-3’

CRMAGE oligo

NT-L-FtsZ To insert ALFA nanotag at G55:Q56 with flanking sequences (GSTLE and LEGST)

5’GCGCCAGCGCCCAGTCCTTTGGTGATACCGCTACCGATTTGAATCGTCTGGGTGCTACCTTCCAGTGCCGTCAGACGACGACGCAGTTCCTGTTCCAGGCCGGATTCCAGGGTGCTACCTCCAACTGCTGTTTTACGCAGCGCTTGTGCATCGGTATTTACCGCGAAGA -3’
